# Population-based study for the comorbidities and associated factors in Ménière’s disease

**DOI:** 10.1038/s41598-022-12492-y

**Published:** 2022-05-18

**Authors:** Min Hee Kim

**Affiliations:** Department of Ophthalmology, Otolaryngology and Dermatology of Korean Medicine, Kyung Hee University Hospital at Gangdong, Dongnam-ro 892, Seoul, 05278 Republic of Korea

**Keywords:** Epidemiology, Endocrine system and metabolic diseases, Immunological disorders

## Abstract

To date, no study has reported the objective metabolic laboratory findings worldwide or the comorbidities for Ménière’s disease (MD) using a population-based design in Asian populations. The aim of this study was to investigate the comorbidities and associated factors for MD using the Korean National Health Insurance Service database. This retrospective population-based study was conducted using a data from the National Sample Cohort database from 2009 to 2015. We only enrolled patients whose records showed a prescription for MD medicine and audiometry findings as well as an appropriate diagnostic code. We also included a matched cohort without MD who were enrolled randomly and matched for sex, age, year of diagnosis, income level, and residential area with the MD group with a ratio of 10:1. We evaluated comorbidities including autoimmune, allergic, metabolic diseases and cancer and the health screening data including general characteristics (height, weight, waist circumference, body mass index, and blood pressure), laboratory findings (fasting glucose, cholesterol, triglyceride, high-density lipoproteintryglyceride (HDL) cholesterol, low-density lipoproteintryglyceride cholesterol, hemoglobin, creatinine, aspartate aminotransferase and alanine aminotransferase, and gamma-glutamyltrans- peptidase (rGT)), and general health behaviors (smoking, alcohol, and exercise) of the MD group, and compared these characteristics with those of the MD-free control group. A total of 2,013 and 20,130 participants were included in the MD and MD-free control groups (1,640 and 15,458 for health screening data). We found the increase in incidence of allergic rhinitis and allergic asthma, decrease in systolic blood pressure, HDL cholesterol, and rGT, and less frequent alcohol consumption and less prevalent smoking in the MD group. No significant differences were observed between the groups in the incidence of autoimmune diseases, and cerebro- and cardiovascular disease as well as health screening data and objective laboratory findings. Inconsistence with published studies, the results of this study suggest that the autoimmunity and metabolic disorder, and skeletal growth might not be associated with the onset of MD. Another well-designed study for other races will be needed to the generalization of this study results.

## Introduction

Ménière’s disease (MD) is a multifactorial disorder of the inner ear characterized by vertigo, tinnitus, and sensorineural hearing loss^1^. Due to changes in diet and an increase in the aging population, the incidence of MD has rapidly increased^2,3^. In recent population-based studies, the incidence of MD was 13–118 per 100,000 persons^2,3^.

MD is a complex heterogeneous disorder, with multiple factors reported to contribute to its development, ^4^ including age (older than 60 years), sex (female) ^2,3^, genetics^5,6^, race (white people)^7^, stature and leg length (short)^8^, metabolic disorder^9,10^, autoimmunity^10–17^, anatomy^18^, allergies^19^, migraines^20^, weather (high humidity and low atmospheric pressure)^21^, diets, and stress^22^. However, the majority of studies are limited by a lack of population-based cohorts and low number of cases; therefore, the underlying disease pathological pathways remain unclear. Furthermore, despite the recent rapid increase in the incidence of MD in Asian populations^23^, few studies have reported the comorbidities of MD in Asian countries.

To the best of our knowledge, to date, no study has reported the objective metabolic laboratory findings worldwide or the comorbidities for MD using a population-based design in Asian populations. The aim of this study was to investigate the comorbidities and associated factors for MD over 7 years in South Korea using the Korean National Health Insurance Service (NHIS) database (Table 1).

**Table 1:** Diagnostic criteria.

	Diagnostic code		Prescription	
	ICD-10	Registration V code	ATC code or ingredients	Days or no. of prescription
**Autoimmune diseases**				
** Rheumatic**				
Systemic lupus erythematosus ^41^	M32	V136		
Dermatomyositis/ polymyositis ^42^	M33	V137		
Systemic sclerosis ^43^	M34	V138		
Sjogren’s syndrome ^44^	M350	V139		
Ankylosing spondylitis ^45^	M45	V140		
Rheumatoid arthritis ^46^	M05	V223		
** Non-rheumatic**				
Type 1 DM	E10		A10 (drugs used in diabetes)	≥ 1 year
Grave’s disease ^47^	E050, E058, E059		Methimazole, propylthiouracil, or carbimazole	≥ 60 days
Hashimoto’s thyroiditis ^47^	E063, E069		Levothyroxine sodium or liothyronine sodium	≥ 60 days
Crohn’s disease ^48^	K50	V130		
Ulcerative colitis ^48^	K51	V131		
Autoimmune hepatitis ^49^	K754	V175		
**Allergic disease**				
Allergic rhinitis	J30		R01 (nasal preparations)	
R06 (Antihistamines for systemic use)	≥ 2 times/year			
Allergic asthma ^50^	J45, J46		R03 (drugs for obstructive airway diseases)	≥ 1 year
**Metabolic disease**				
Cerebrovascular	–	V191		
Cardiovascular	–	V192		
**Cancer**	–	V193, V194		

ATC, Anatomical Therapeutic Chemical; DM, diabetes mellitus.

## Result

### Baseline characteristics of participants

Finally, 2,013 participants with MD and 20,130 controls (1,640 and 15,458 for health screening data) were enrolled in the analysis. No significant differences in age, sex, year of diagnosis, income level, and residential area were observed between the two cohorts (Table 2).

**Table 2:** Baseline characteristics of participants.

Variable	Comorbidity data			Health screening data		
	MD (n = 2,013)	Control (n = 20,130)	*P* value	MD (n = 1,640)	Control (n = 15,458)	*P* value
**Age (years)**						
Mean (SD)	49.0 (16.5)	48.9 (16.5)	0.970	52.3 (14.2)	52.0 (14.3)	0.547
**Sex, n (%)**			0.930			0.780
Male	661	6585		545	5079	
Female	1352	13,545		1095	10,379	
**Diagnosis year**			1.000			0.997
2008	59	590		52	468	
2009	53	530		44	427	
2010	105	1050		96	847	
2011	130	1300		110	1011	
2012	231	2310		185	1779	
2013	316	3160		251	2432	
2014	479	4790		386	3621	
2015	640	6400		516	4873	
**Income level**			1.000			0.984
1	138	1390		112	1056	
2	141	1394		118	1085	
3	145	1425		123	1081	
4	144	1434		122	1086	
5	159	1596		134	1237	
6	163	1653		136	1274	
7	193	1936		153	1576	
8	240	2394		201	1885	
9	264	2677		211	2105	
10	331	3354		278	2607	
NA	95	877		52	466	
**Regional area**			0.887			0.888
Seoul	476	23.81		388	3553	
Busan	160	8.05		132	1256	
Daegu	81	3.93		65	617	
Incheon	91	4.52		70	692	
Gwangju	130	6.30		106	990	
Daejeon	62	2.99		49	467	
Ulsan	20	1.00		19	168	
Sejong	3	0.03		3	5	
Gyeonggi-do	408	20.36		335	3127	
Gangwon-do	72	3.69		56	549	
Chungcheongbuk-do	34	1.63		27	274	
Chungcheongnam-do	80	4.12		68	664	
Jeollabuk-do	24	1.20		18	179	
Jeollanam-do	178	8.93		149	1411	
Gyeongsangbuk-do	37	1.95		28	311	
Gyeongsangnam-do	135	6.74		112	1083	
Jeju-do	22	0.76		15	112	

*MD* Ménière disease.

### Comorbidities associated with MD

Of the autoimmune diseases, we found no significant association between MD and rheumatoid arthritis, ankylosing spondylitis, type 1 diabetes mellitus (DM), Grave’s disease, and Hashimoto’s thyroiditis. Due to the limited number of patients with each autoimmune disease, we also compared the total number of autoimmune rheumatic diseases (systemic lupus erythematous, dermatomyositis/polymyositis, systemic sclerosis, Sjogren’s syndrome, ankylosing spondylitis, and rheumatoid arthritis) and autoimmune non-rheumatic diseases (type 1 DM, Grave’s disease, Hashimoto’s thyroiditis, Crohn’s disease, ulcerative colitis, autoimmune hepatitis), and no significant differences were observed between the two groups. Among the allergic diseases, the patients with MD were at increased risk of allergic rhinitis (odds ratio [OR] 2.000, 95% CI 1.790–2.240) and allergic asthma (OR 1.469, 95% CI 0.992–2.101) compared with the controls. Regarding the incidence of cerebro- and cardiovascular diseases and cancer, there were no significant differences between the two groups (Table 3).

**Table 3:** Comorbidities of participants with Ménière’s disease.

	Prevalence rate per 1,000 (n)		Univariable analysis	
	MD	Controls without MD	Adjusted OR (95% CI)	*P* Value
**Autoimmune diseases**				
** Rheumatic**				
Rheumatoid arthritis	2.48 (5)	4.22 (85)	0.587 (0.207–1.307)	0.248
Ankylosing spondylitis	1.49 (3)	1.14 (23)	1.305 (0.309–3.752)	0.665
Total^a^	0.35 (8)	0.64 (129)	0.541 (0.229–1.075)	0.114
** Non-rheumatic**				
Type 1 DM	1.49 (3)	3.87 (78)	0.384 (0.094–1.027)	0.104
Grave’s disease	3.97 (8)	4.37 (88)	0.909 (0.405–1.761)	0.796
Hashimoto’s thyroiditis	6.46 (13)	4.77 (96)	1.356 (0.723–2.335)	0.304
Total^b^	1.44 (29)	1.43 (288)	1.007 (0.671–0.971)	0.971
** Allergic disease**				
Allergic rhinitis	774.47 (1599)	658.82 (6652)	2.000 (1.790–2.240)	< 0.001**
Allergic asthma	15.90 (32)	10.88 (103)	1.469 (0.992–2.101)	0.044*
** Metabolic disease**				
Cerebrovascular	5.96 (12)	5.27 (106)	1.133 (0.590–1.977)	0.683
Cardiovascular	14.90 (30)	14.21 (286)	1.050 (0.704–1.507)	0.802
**Cancer**	6.06 (122)	5.68 (572)	1.034 (0.849–1.248)	0.732
**Migraine**	229.01 (461)	69.05 (1390)	4.005 (3.559–4.500)	< 0.001**

Significant values are in [bold].

^a^Systemic lupus erythematous, Dermatomyositis/polymyositis, systemic sclerosis, Sjogren’s syndrome, ankylosing spondylitis, rheumatoid arthritis.

^b^ Type 1 diabetes mellitus, grave’s disease, hashimoto’s thyroiditis, crohn’s disease, ulcerative colitis, autoimmune hepatitis.

*MD* Ménière’s disease, *DM* Diabetes mellitus.

### Health screening data

Among the general characteristics studied, we found no significant differences in height, weight, body mass index (BMI), and waist circumference between the patients with MD and controls. Systolic BP was significantly lower in the patients with MD. Laboratory findings showed that HDL cholesterol and rGT levels significantly decreased in the patients with MD. We found no significant differences in fasting glucose, total cholesterol, triglyceride, LDL cholesterol, hemoglobin, creatinine, AST, and ALT levels between the patients with MD and controls. Of the general health behaviors, alcohol intake and smoking history were less frequent in the patients with MD, whereas there were no differences in exercise frequency (Table 4).

**Table 4:** Health screening data of participants with Ménière’s disease.

	MD	Controls without MD	*P* value
**General characteristics**			
Height, mean (SD), cm	160.89 (8.8)	160.90 (8.83)	0.948
Weight, mean (SD), kg	61.24 (10.75)	61.48 (11.39)	0.391
Body Mass Index, mean (SD), kg/m2	23.58 (3.11)	23.67 (3.4)	0.292
Waist circumference, mean (SD), cm	79.25 (9.21)	79.4 (9.51)	0.550
Systolic blood pressure, mean (SD), mmHg	121.01 (14.89)	122.01 (15.78)	0.010*
Diastolic blood pressure, mean (SD), mmHg	75.13 (9.96)	75.63 (10.13)	0.054
**Laboratory findings**			
Fasting glucose, mean (SD), mg/dL	97.95 (21.43)	98.7 (24.33)	0.185
Total cholesterol, mean (SD), mg/dL	196.12 (37.14)	195.78 (37.95)	0.726
Triglyceride, mean (SD), mg/dL	124.36 (83.15)	127.11 (112.47)	0.246
HDL cholesterol, mean (SD), mg/dL	56.05 (14.22)	57.04 (22.33)	0.018*
LDL cholesterol, mean (SD), mg/dL	115.72 (43.48)	115.09 (42.92)	0.597
Hemoglobin, mean (SD), g/dL	13.58 (1.50)	13.55 (1.58)	0.465
Creatinine, mean (SD), mg/dL	0.86 (0.46)	0.87 (0.54)	0.501
AST, mean (SD), U/L	24.93 (13.81)	25.02 (16.45)	0.801
ALT, mean (SD), U/L	24.07 (22.04)	23.41 (20.88)	0.244
rGT, mean (SD), U/L	29.94 (32.95)	32.94 (48.34)	< 0.001**
**Smoking history**			< 0.001**
Never, No. (%)	1101 (74.75)	9870 (72.46)	
Former, No. (%)	202 (13.71)	1620 (11.89)	
Current, No. (%)	168 (11.41)	2119 915.56)	
N/A, No. (%)	2 (0.14)	12 (0.09)	
**Alcohol intake**			< 0.001**
Never, No. (%)	930 (63.14)	8512 (62.49)	
1 times/wk, No. (%)	161 (10.93)	1693 (12.43)	
2 times/wk, No. (%)	126 (8.55)	1222 (8.97)	
3 times/wk, No. (%)	108 (7.33)	926 (6.80)	
4 times/wk, No. (%)	44 (2.99)	394 (2.89)	
5 times/wk, No. (%)	41 (2.78)	418 (3.07)	
6 times/wk, No. (%)	25 (1.70)	173 (1.27)	
Daily, No. (%)	35 (2.38)	261 (1.92)	
N/A, No. (%)	6 (0.41)	22 (0.16)	
**Exercise**^**a**^			0.628
Never, No. (%)	810 (54.99)	7488 (54.97)	
1 times/wk, No. (%)	165 (11.20)	1730 (12.70)	
2 times/wk, No. (%)	158 (10.73)	1450 (10.65)	
3 times/wk, No. (%)	142 (9.64)	1221 (8.96)	
4 times/wk, No. (%)	61 (4.14)	519 (3.81)	
5 times/wk, No. (%)	57 (3.87)	548 (4.02)	
6 times/wk, No. (%)	32 (2.17)	231 (1.70)	
Daily, No. (%)	45 (3.05)	410 (3.01)	
N/A, No. (%)	3 (0.20)	24 (0.18)	

Significant values are in [bold].

^a^30 min or more of moderate-intensity exercise.

*MD* Ménière’s disease, *LDL* Low-density lipoprotein, *HDL* High-density lipoprotein, *AST* Aspartate aminotransferase, *ALT* Alanine aminotransferase, *rGT* Gamma-glutamyltrans- peptidase.

## Discussion

In this large population-based study, we investigated comorbidities including autoimmune, allergic and metabolic diseases, cancer, as well as health screening data (including general characteristics, laboratory findings, and general health behaviors of patients with MD), and compared these characteristics to those of matched MD-free controls.

We found an increase in the incidence of allergic rhinitis and allergic asthma in patients with MD, similar to that in previous studies that reported a higher number of patients with a history of allergy and the prevalence of allergic diseases, a higher positive rate of the allergic skin test, and elevated Immunoglobulin E levels in patients with MD compared with those in controls^3,24–26^. Randomized controlled studies regarding the treatment of allergy and its association with MD attacks would be helpful to achieve confirmative conclusions.

We further found less frequent alcohol consumption and less prevalent smoking in patients with MD, consistent with the findings of previous studies that reported a lower OR in current smokers and alcohol drinkers^10^ and low prevalence of smoking and alcohol consumption among MD cases^3^. For alcohol consumption in patients with MD, there are positive aspects, such as alcohol consumption has inhibitory effects on hypothalamic vasopressin production, leading to an increase of diuresis and reduction of endolymphatic pressure^27^. This might be a possible cause for less frequent alcohol consumption in the MD population. However, there is a negative aspect that alcohol has toxic effects on the cochlear and labyrinth^28^, therefore, clinicians should not guide their patients to consume more alcohol before more confirmatory studies are conducted. There are no studies that reported a direct association between smoking and MD; however, there is a retrospective study that reported that smoking cessation contributes to the prevention of new peripheral vestibular disorders among males^29^. Further, smoking is known to cause vasoconstriction and a decrease in blood flow. Since alcohol consumption and smoking have a positive correlation^30^, alcohol consumption might be a confounder in the association between smoking and MD.

Moreover, no significant differences in the incidence of autoimmune diseases and cerebro- and cardiovascular disease were observed between patients with MD and controls. In health screening data, body weight, waist circumference, BMI, and objective laboratory findings (fasting glucose, cholesterol, triglyceride, LDL cholesterol, AST, and ALT levels) were not significantly different between patients with MD and controls. Moreover, systolic BP, HDL cholesterol, and rGT levels were lower in patients with MD.

The exact mechanism of MD remains unclear, although several etiologies including autoimmune, allergic, genetic, traumatic, or infectious (viral) conditions have been proposed^1^. The association between autoimmunity and MD has been supported by the high prevalence of systemic autoimmune diseases in patients with MD^10,13–15^. The relationship between metabolic disorder and MD has also been suggested. Previous studies have reported that DM is associated with severe hearing loss and frequent vertigo in cases of MD^9^ and higher BMI and systolic BP are related to MD^10^. Therefore, the present study results are not consistent with those of previous studies.

The primary disadvantage of administrative database-based research is the risk of inaccurate cohort identification, which can lead to selection bias. Therefore, definitions of specific diseases determine the quality of the population-based study^31^. Previous studies used the definition of diseases only based on diagnostic code, not including specific tests or treatments, used self-reported diagnosis, or did not match the control cohort with patient cohort^10,13–15^. In this study, MD was defined not only based on diagnostic code but also based on the audiometry findings and medications^2^. Moreover, to select accurate comorbidities, we applied a registration program code that requires many tests or prescription history or used prescription history, refers to the previous studies. Therefore, selection bias might be the reason for the heterogeneity between the results of previous studies and this study. However, elevated levels of serum autoantibodies^16,17^, cytokines, and chemokines^12^ in patients with MD have also been reported in Italian and Spain studies. Therefore, there is a possibility that the lack of significance in autoimmune diseases might be due to racial characteristics.

A previous UK study reported that tall stature and high leg length are associated with the prevalence of MD, suggesting that early life environmental exposures may influence skeletal growth and onset of MD^8^. However, inconsistent with the previous study, height was not significantly different between the two groups in this study. This may be due to the self-reported diagnosis of MD in the previous study^8^ or racial differences.

In conclusion, this study observed the comorbidities and conditions of patients with MD using a national population database, multiple diagnostic criteria, and stratified matched control group. To the best of our knowledge, this study is the first globally to observe objective laboratory findings and to compare height, weight, BMI, waist circumference, BP, and many comorbidities using population-based design in Asian countries. Some of the results are not consistent with those of previous studies; therefore, other well-designed studies for other races will be needed to generalize the results of this study.

## Conclusions

We investigated the comorbidities including autoimmune, allergic, metabolic diseases and cancer and the health screening data including general characteristics (height, weight, waist circumference, body mass index, and blood pressure), laboratory findings (fasting glucose, cholesterol, triglyceride, HDL cholesterol, low-density LDL cholesterol, hemoglobin, creatinine, AST, ALT, and rGT), and general health behaviors (smoking, alcohol, and exercise) of patients with MD using a national population database. We found the increase in incidence of allergic rhinitis and allergic asthma, decrease in systolic blood pressure, HDL cholesterol, and rGT, and less frequent alcohol consumption and less prevalent smoking in the MD group. Inconsistence with published studies, the results of this study suggest that the autoimmunity and metabolic disorder, and skeletal growth might not be associated with the onset of MD. In this study, we applied a registration program code, used multiple diagnostic criteria, and stratified matched control group to minimize the selection bias; however, previous studies used the definition of diseases only based on diagnostic code, not including specific tests or treatments, used self-reported diagnosis, or did not match the control cohort with patient cohort. Therefore, selection bias might be the reason for the heterogeneity between the results of previous studies and this study. Another well-designed study for other races will be needed to the generalization of this study results.

## Methods

### Data source

In South Korea, 97% of the population is covered by national health insurance, and the remaining 3% is covered by the medical aid program. The Korean NHIS includes all claims of both these programs; thus, it can be used as a data source to identify the health status of the whole nation. Using the sub-datasets, we selected a National Sample Cohort (NSC) database (DB), which has been used in previous studies^32,33^. The NHIS-NSC DB is an approximately 2% random sample (n = 1,000,000) of all citizens stratified according to age, sex, income level, and residential area. The DB includes data on the participants’ insurance eligibility, medical treatment history, health care provider’s institution, and general health screening data from 2002 to 2015. In this study, we used NHIS-NSC data from 2009 to 2015. A more detailed description of these data is provided in a previous study^34^. All methods were performed in accordance with the relevant guidelines and were approved by the Institutional Review Board (IRB) of the Kyung Hee University Hospital at Gangdong (IRB No.2020-03-007). Informed consent was unnecessary because this study involved minimal risk to human subjects, and its requirement was waived by the IRB of the Kyung Hee University Hospital at Gangdong (IRB No.2020-03-007).

### Study population

In the present study, a patient with MD was operationally defined as a person who had a diagnostic code of MD (International Classification of Diseases 10th revision [ICD-10] codes: H810 or H810.002) as the primary or secondary diagnosis code, had a record of pure-tone audiometry (codes E6931 and F6341), and who had been prescribed betahistine, referring to the diagnostic criteria established by the American Academy of Otolaryngology-Head and Neck Surgery^35,36^ and Barany Society^37^ and previous UK and Korea nationwide population-based study of MD^2,3^ (washout period was from 2002 to 2004). As in other previous population-based studies, because of the limitation of the administrative database, the degree or type of hearing loss and the frequency of vertigo could not be applied to the definition of the disease. A matched cohort without MD was enrolled randomly by matching patients by sex, age, year of diagnosis, income level, and residential area to the MD group at a ratio of 10:1.

### Comorbidities based on medical treatment history

The South Korean government has enhanced benefit coverage and established a registration program for four major conditions (cancer, cardiovascular disease, cerebrovascular disease, and rare diseases). The patients must meet the diagnostic criteria related to the imaging, cultivation, genetic, histologic test, or clinical diagnosis, which is applied differently to each condition, to be registered in this program. In this study, comorbidity data were extracted using the V code from the registration program or prescription history combined with ICD-10 diagnosis code (comorbidities that cannot apply V code) to identify patients more accurately with comorbidities. We evaluated four types of comorbidities: (i) autoimmune diseases (rheumatic and non-rheumatic), (ii) allergic diseases, (iii) metabolic diseases, and (iv) cancer. The details of the diagnostic criteria are presented in Table 1. We included the comorbidities which could refer to the definition of disease according to the previous population based Korean studies. We also referred to the comorbidities in the other disorder in previous population based Korean studies^38,39^.

### Health screening data

All insured Koreans older than 40 years undergo a biannual health checkup supported by the NHIS, and employees older than 20 years are required to undergo health checkups once a year. We used general health screening data between 2009 and 2015 in the NHIS-NSC DB. Regarding general health screening data, weight, height, waist circumference, and blood pressure (BP) were measured. Fasting blood glucose, total cholesterol, triglyceride, high-density lipoprotein (HDL) cholesterol, low-density lipoprotein (LDL) cholesterol, hemoglobin, serum creatinine, aspartate aminotransferase (AST), alanine aminotransferase (ALT), and gamma-glutamyl transferase (rGT) levels were also measured in a fasting state. Information on general health behaviors such as alcohol consumption, smoking, and exercise was obtained using self-report questionnaires^40^.

### Statistical analysis

We evaluated associations between MD and comorbidities using univariate logistic regression after adjusting for age, sex, and socioeconomic status. Continuous variables are expressed as number of participants analyzed, mean, standard deviation, median, minimum, and maximum values and categorical variables as frequency and percentage. All statistical analyses were performed using R version 3.5.1. (Foundation for Statistical Computing, Vienna, Austria).

## Data Availability

The datasets used and/or analyzed during the current study available from the corresponding author on reasonable request.
